# Evaluation of Attachment Styles in Patients with Chronic Pruritus

**DOI:** 10.3390/jcm15062167

**Published:** 2026-03-12

**Authors:** Kıvılcım Çınkır Özsaraç, Şadiye Visal Buturak, Deniz Öztürk Kara, Özgür Gündüz, Ayşe İşcan Özdemir, Mehtap Kıdır

**Affiliations:** 1 Department of Dermatology, Kırıkkale University Faculty of Medicine, Kırıkkale 71450, Turkey; drdenizoz@gmail.com (D.Ö.K.); gunduzozgur@windowslive.com (Ö.G.); 2 Department of Psychiatry, Kırıkkale University Faculty of Medicine, Kırıkkale 71450, Turkey; visalbuturak@hotmail.com; 3 Department of Dermatology, Kayseri Education and Research Hospital, Kayseri 38030, Turkey; ayseiscan22@hotmail.com; 4 Department of Dermatology, Dumlupınar University Faculty of Medicine, Kütahya 43100, Turkey; mehtap.kidir@gmail.com

**Keywords:** chronic pruritus, attachment, insecure attachment, psychopathology, psychodermatology

## Abstract

**Background and Objectives:** While associations between attachment styles and certain dermatologic conditions have been documented, their role in chronic pruritus remains unexplored. Given the significant psychosomatic component in the etiology of chronic pruritus, this study aimed to assess attachment styles in patients with chronic pruritus in the absence of organic or psychiatric disorders and to examine their potential contribution to its development. **Methods:** Sixty patients with chronic pruritus were compared with a healthy control group (*n* = 60). Socio-demographic data, the duration of the disease, and the itch severity were noted. Additionally, assessments performed via the Questionnaire of Relation Scale, Questionnaire of Relation, Hospital Anxiety and Depression Scale, General Health Questionnaire, and Dermatology Life Quality Index (DLQI). **Results:** Statistically higher scores of fearful, dismissive, and preoccupied attachment styles were observed in the patient group compared to the control group. Among patients, those with moderate to high itch severity had higher mean scores of anxiety and preoccupied attachment than those with low itch severity. In contrast, secure attachment scores were significantly higher in the control group than in the patient group. Limitations: Attachment styles were examined with a self-report instrument without stimulated recall procedures. **Conclusions:** Our findings clearly demonstrate that patients with chronic pruritus exhibit significantly higher levels of insecure attachment styles alongside elevated anxiety, depression, and psychosocial burden. Notably, the association between preoccupied attachment and greater itch severity highlights how emotional dysregulation may intensify pruritus symptoms. Due to limited research directly examining attachment in chronic pruritus, our study provides novel insight and supports a biopsychosocial approach to care.

## 1. Introduction

Pruritus, an unpleasant sensation that leads to scratching, is one of the most common symptoms in dermatologic diseases [1]. It has been documented that, in cases of chronic pruritus lasting more than six weeks with no identifiable dermatologic cause, itch may present as a somatic manifestation of underlying psychiatric conditions such as depression and anxiety. Furthermore, chronic pruritus can have a significant negative impact on quality of life [2,3].

**Figure 1:**
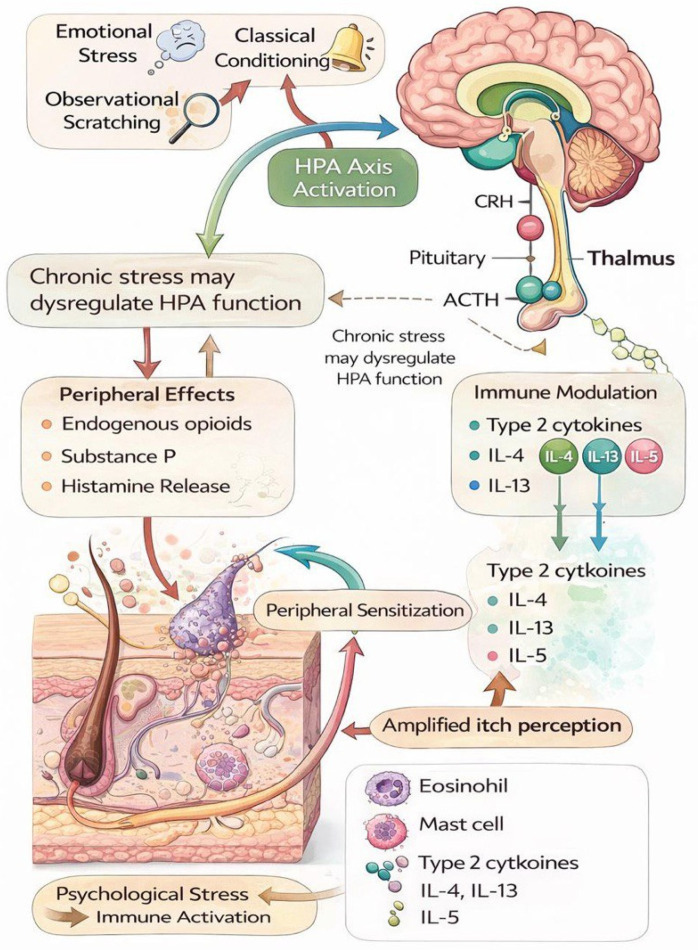
Psychoneuroimmune mechanisms involved in chronic pruritus. Psychological stress and conditioned responses activate the hypothalamic-pitutary-adrenal (HPA) axis, leading to neuroendocrine mediator release. Chronic stress may dysregulate HPA function and promote the release of endogenous opioids, histamine, and substance P. In paraliel, type 2 cytokines-including IL-4, IL-13, and IL-5 contribute to eosinophil-mediated inflammation and penpheral sensitiza.

The skin and brain share a common embryonic origin from the neuroectoderm [4]. Evidence for a psychogenic component of itch arises from studies showing that histamine release can be triggered in guinea pigs through stress induced by classical conditioning and that patients with atopic dermatitis experience increased itch when observing others scratching [5,6]. Several studies have demonstrated a direct link between pruritus and stress [7]. Suppression of the hypothalamic–pituitary-adrenal (HPA) axis and the release of mediators such as endogenous opioids may trigger scratching behavior under stress [8]. Chronic stress can also promote itch through the activation of histamine, vasoactive amines, and substance P [9]. Beyond classical mediators such as histamine and substance P, increasing evidence indicates inflammatory cytokines such as IL-5 play a substantial role in chronic inflammatory and pruritic conditions [10]. These mechanisms support a biopsychosocial framework in which immune activation and psychological vulnerability jointly modulate chronic itch (Figure 1).

Attachment theory describes an emotional bond that originates in early caregiver–child relationships and persists throughout life. Attachment patterns tend to become more pronounced under chronic stress [11,12]. Bartholomew’s four-category model—secure, preoccupied, dismissing, and fearful—is based on two dimensions: the positivity or negativity of an individual’s self-model. A negative self-perception forms the basis of insecure attachment [13].

Insecure attachment styles—preoccupied, dismissing, and fearful—may contribute to skin diseases through stress-related mechanisms involving impaired HPA axis function, reduced cortisol, and elevated inflammatory cytokines [14,15]. Associations between attachment patterns and conditions such as atopic dermatitis, chronic urticaria, vitiligo, and plaque psoriasis have been well-documented [16,17,18,19]. However, studies directly linking attachment styles to pruritus remain limited. Therefore, we aimed to evaluate attachment styles in patients with chronic pruritus in the absence of organic or psychiatric causes and to explore potential etiologic and predisposing roles of attachment in its development.

## 2. Materials and Methods

### 2.1. Sample

This study was performed in the Dermatology Department of Kırıkkale University Medical School between February 2014 and May 2014. The study was conducted in accordance with the Declaration of Helsinki and approved by the Kırıkkale University Clinical Research Ethics Committee (approval number: 03/02; date: 28 January 2014). Data were collected in 2014 within the framework of this ethically approved protocol, and the present analysis was conducted retrospectively in accordance with the original study design. Sixty patients diagnosed with chronic pruritus were compared with a healthy control group matched for age, gender, and educational level (*n* = 60) and without any skin disease. Participants were enrolled in this study after providing written informed consent.

Inclusion criteria for this study were age above 18 years and being diagnosed with chronic pruritus without any possible etiologic explanation. Exclusion criteria included the presence of dermatologic, systemic, metabolic, allergic, endocrine (e.g., thyroid disease), hepatic, renal, diabetic, or psychiatric conditions known to be associated with pruritus. All participants were evaluated through clinical examination and review of medical records to exclude identifiable organic causes.

### 2.2. Measures

Socio-demographic questionnaire: Includes socio-demographic data such as age, gender, educational state, occupation, marital state, and place of residency as well as information related to the childhood of the participant.

General Health Questionnaire (GHQ): Developed by David Goldberg for recognition of mental disorders in primary health care practice. In our study, we used GHQ consisting of 12 items, and the scoring was carried out according to the Goldberg method. The cutoff point for the scale was accepted as 2 [20].

Dermatology Life Quality Index (DLQI): A practical questionnaire in order to determine impact of dermatological diseases on an individual’s quality of life. It consists of 11 items, with each item scored between 0 to 4, and takes the last one month into consideration. Additionally, the scale has psychosocial and physical parts [21].

Hospital Anxiety and Depression Scale (HADS): Introduced by Zigmond and Snaith for the assessment of anxiety and depression risks in outpatients and to measure its level and severity. The scale consists of fourteen items; seven of them (the odd-numbered items) measure anxiety, while the remaining seven (even-numbered items) measure depression. The cut off points are 10/11 for the anxiety subscale and 7/8 for the depression subscale [22].

Itch Severity Scale: In the present study, itch severity was assessed exclusively using the Itch Severity Scale; a visual analog scale (VAS) was not employed. It was developed by Majeski et al. specifically for assessment of how and the severity with which the itch is perceived by the patient; its reliability for internal consistency and test–retest validity is quite high and consists of 7 items [23,24].

Questionnaire of Relation Scale (QRS): Designed by Bartholomew and Horowitz in order to measure four attachment styles and consists of 30 items. Participants are asked to rate their degree of correspondence to each prototype on a 7-point scale (1= does not describe me; 7= totally describes me) [13].

Questionnaire of Relation (QR): Developed by Bartholomew and Horowitz. It consists of four short paragraphs associated with four attachment types. Participants are asked to rate their degree of correspondence to each prototype on a 7-point scale [13,14,15,16,17,18,19,20,21,22,23,24,25].

### 2.3. Statistical Analysis

All data were analyzed using SPSS for Windows (version 21.0; IBM Corp., Armonk, NY, USA). Descriptive statistics—including means, standard deviations, medians (min–max), frequency distributions, and percentages—were used to summarize the data. The normality of data distribution was assessed using the Kolmogorov–Smirnov test. For normally distributed variables, comparisons between two groups were conducted using the Student’s *t*-test. For variables that were not normally distributed, the Mann–Whitney U test and Kruskal–Wallis test were applied to compare differences between groups. Categorical variables were evaluated using the chi-square test. The presence of correlation was analyzed with Spearman’s rho and Pearson tests. *p*-values of <0.05 were considered statistically significant.

## 3. Results

There were no statistically significant differences between the chronic pruritus group (mean age: 44.60 ± 15.25 years; 71.7% female, 28.3% male) and the control group (mean age: 44.25 ± 14.80 years; 73.3% female, 26.7% male) in terms of age (*p* = 0.899) or gender distribution (*p* = 0.838); however, the rate of unemployment was significantly higher in the patient group (65.0%) compared to controls (34.4%) (*p* < 0.0001). The mean duration of disease was 29.58 ± 3.92 months (range: 2–180 months) in patients with chronic pruritus (Table 1).

We classified itchiness severity as low if below the median value (8.25) or as moderate/severe if above the median value (range of scoring: 0–21) [23]. DLQI, GHQ, and HADS—anxiety scores in patients with moderate/severe itchiness were statistically higher (respectively: *p* < 0.0001, *p* = 0.003, and *p* = 0.003) when compared to patients with low itchiness (Table 2).

Data related to attachment styles with pruritus:

The mean score for preoccupied attachment style in patients with moderate/severe itchiness severity was found statistically higher than patients with low itchiness severity. Moreover, there were no statistically significant differences between average scores of other attachment types and the severity of itchiness (Table 3). In addition, there was no statistically significant difference between the severity of itchiness and the mean score for perception of self and others.

Average scores for fearful, dismissive, and preoccupied attachment styles were found statistically higher in the patient group than the control group. On the other hand, the secure attachment mean score was statistically higher in the control group than the patient group (Table 4). We observed a negative, moderate, and significant correlation between anxiety and depression scores with only secure attachment type in patients (respectively: r = −0.32, *p* = 0.011; r = −0.335, *p* = 0.009).

Data related to psychopathology and pruritus:

The total mean scores of GHQ, HAD—anxiety, HAD—depression, and DLQI were found significantly higher in the patient group than the control group (Table 5). When we analyzed the Relation Questionnaire, the average score for self-perception in the patient group was found statistically lower than in the patient group (*p* ≤ 0.001), and the average score for the perception of others was found statistically higher (*p* = 0.03) in the control group than in the patient group.

The average scores for attachment types were compared with the average scores for self-perception and the perception of others in our study. There were negative–low, negative–moderate, negative–moderate, and positive–moderate statistically significant correlations detected between the score for patients’ self-perception and dismissive, fearful, preoccupied, and secure attachment style scores, respectively. In the control group, a negative-low correlation was detected between scores of self-perception and fearful attachment styles only.

A negative–moderate correlation was observed between the score for the perception of others and the score for fearful and dismissive attachment styles in patients. In addition, a positive–moderate correlation was detected between the score for the perception of others and the score for secure attachment style in the control group (Table 6).

## 4. Discussion

An increasing number of studies highlight the significant role of psychosocial and psychiatric contributors in the persistence and intensity of chronic pruritus, emphasizing the importance of exploring deeper psychological dimensions such as attachment styles in affected individuals [26]. In addition, recent research has shown that chronic pruritus frequently presents in middle-aged adults and tends to affect females more than males. Pereira et al. analyzed a cohort of 263 patients with chronic pruritus of unknown origin and reported a median age of 55 years, with females comprising 58.6% of the sample and males 41.4%. The condition was reported to persist for over a year in 77.6% of patients [27]. These demographic patterns are largely consistent with our findings, which revealed a mean age of 44.60 years and a female predominance of 71.7% in the chronic pruritus group.

Published data have highlighted the considerable psychosocial burden faced by patients with chronic pruritus, often manifesting in emotional distress, reduced functionality, and impaired social engagement. In a large-scale cross-sectional study conducted across nine European centers (*n* = 552), Steinke et al. observed that patients with chronic pruritus experienced significant reductions in quality of life, which were closely linked to both the intensity of pruritus and broader psychosocial impacts such as disrupted daily life and occupational dysfunction [28]. Similarly, Zeidler et al. found that individuals with chronic prurigo, a severe form of chronic pruritus, reported higher disease burden scores compared to patients with non-lesional pruritus, including limitations in employment and social roles [29]. Consistently, our study identified a significantly higher rate of unemployment among patients with chronic pruritus (65.0%) compared to healthy controls (34.4%).

Quality of life impairment is a well-established feature of chronic pruritus, with several studies indicating a direct association between itch severity and dermatological quality of life scores. In a large-scale analysis of 1150 patients with various types of chronic pruritus, Stumpf et al. demonstrated that both the DLQI and the pruritus-specific ItchyQoL scores were significantly elevated, particularly in patients reporting higher itch intensity. The study confirmed that the DLQI, despite being more general than ItchyQoL, remains a valid tool to assess life-quality impairment in these patients. Mean DLQI scores in this cohort were strongly correlated with itch symptoms and emotional distress [30]. In accordance with these data, DLQI scores were significantly higher in the patient group than in the healthy controls in the present study. Furthermore, our study also revealed significantly higher DLQI scores among patients experiencing moderate to severe pruritus, supporting the conclusion that itch intensity is a key determinant of reduced quality of life in chronic pruritus.

Mounting clinical evidence underscores a compelling link between chronic pruritus and underlying psychiatric disturbances, particularly anxiety and depressive symptoms. In a large-scale longitudinal study involving patients from the University Hospital Münster’s Center for Chronic Pruritus, Schneider et al. demonstrated that individuals suffering from chronic pruritus with psychiatric or psychosomatic comorbidities showed significantly elevated scores on the HADS—anxiety and HADS—depression scales, which closely correlated with itch intensity and overall disease burden [31]. Similarly, Boehlig et al. reported that among 123 patients with nonalcoholic fatty liver disease, those with moderate to severe pruritus had a 12% prevalence of anxiety and 4% prevalence of depression (measured by HADS), with anxiety showing a statistically significant association with pruritus severity (OR = 7.75, *p* = 0.011) [32]. Supporting this, our findings revealed that the total mean scores for GHQ (3.98 vs. 1.20, *p* < 0.0001), HADS—anxiety (8.23 vs. 6.00, *p* = 0.006), and HADS—depression (8.03 vs. 4.95, *p* < 0.0001) were all significantly higher in the patient group than in healthy controls. Moreover, our data revealed that patients with moderate to severe itch reported significantly higher levels of psychological distress, as reflected in their GHQ and HADS—anxiety scores (*p* = 0.003 for both), reinforcing the robust association between itch severity and emotional dysfunction.

Despite the growing recognition of psychological factors in dermatological disorders, direct research evaluating the role of adult attachment styles in chronic pruritus remains notably limited. While attachment theory has been widely applied in the context of psychosomatic conditions, its specific relevance to chronic itch is only beginning to be explored. One recent example is the population-based study by Stamp et al., which reported that individuals with a fearful attachment style were more than twice as likely to experience chronic pain syndromes—including chronic pruritus—compared to those with secure attachment (fearful: 49%; secure: 23%; OR = 2.95, *p* < 0.001) in a large population-based survey (*n* = 2371). All three insecure attachment styles—fearful, preoccupied, and dismissive—were significantly associated with chronic pruritus prevalence [33]. However, such studies are still rare, and the majority of available evidence stems from dermatological populations with similarly psychogenic components. Szabo et al., in a multicenter European study including 3635 dermatological outpatients and 1359 controls, demonstrated that patients with skin diseases reported significantly lower levels of comfort with closeness and dependence, consistent with insecure attachment styles [34]. Securely attached individuals were less likely to report recent stressful life events, and patient satisfaction with medical care was higher among those with secure attachment profiles [16]. These results align with prior evidence from dermatological subgroups; Dieris-Hirche et al. observed that 62 adult patients with atopic dermatitis showed significantly higher levels of insecure attachment compared to healthy controls [16]. These outcomes are consistent with our findings, which revealed that patients with chronic pruritus exhibited significantly higher scores for fearful (*p* < 0.001), dismissive (*p* = 0.041), and preoccupied (*p* = 0.048) attachment styles, while secure attachment scores were lower compared to the control group (*p* < 0.001). Furthermore, when stratified by itch severity, only preoccupied attachment style showed a statistically significant elevation in the moderate/severe itch subgroup compared to those with milder symptoms (*p* = 0.01), indicating that this particular form of relational insecurity may be more sensitive to symptom intensity. This observation aligns with previous research linking preoccupied attachment with heightened emotional dysregulation and somatic amplification and may reflect a maladaptive attentional focus on bodily discomfort in response to a perceived interpersonal threat or distress [35].

Moreover, we also aimed to evaluate the relationship between attachment styles and psychosomatic factors along with the relationship between chronic pruritus–attachment theory and measurement of anxiety/depression score. Accordingly, we found a negative relationship between secure attachment style and anxiety–depression scores and a positive relationship between preoccupied attachment style and anxiety. Recent researches highlighted that major depression is associated with insecure attachment type (especially fearful and preoccupied), and depressive symptoms diminish with secure attachment. In a complementary study, Demirci et al. focused on patients with psoriasis and found that insecure attachment styles—especially anxious/ambivalent and avoidant—were positively correlated with higher levels of depression, anxiety, and reduced quality of life (measured by HADS and DLQI), even though overall attachment style distributions were not significantly different from controls. These findings provide compelling evidence that insecure attachment may contribute to psychological distress and maladaptive coping in dermatological populations [36].

The internal working models of self and others, central to Bartholomew and Horowitz’s four-category attachment theory, offer a valuable lens through which to interpret patterns of relational insecurity in chronic pruritus patients [13]. In our study, self-perception scores were significantly lower (*p* ≤ 0.001) and perception of others scores significantly higher (*p* = 0.03) in the control group compared to the patient group, suggesting a more negative internal representation of self among patients. Furthermore, correlation analyses demonstrated that among chronic pruritus patients, self-perception scores were negatively associated with dismissive (r = −0.269, *p* = 0.04), fearful (r = −0.492, *p* < 0.0001), and preoccupied (r = −0.315, *p* = 0.01) attachment styles, while a positive correlation was observed with secure attachment (r = 0.37, *p* = 0.005). These findings align with the Bartholomew model, which posits that individuals with insecure attachment—especially fearful and preoccupied types—harbor negative views of the self and, in some cases, of others. Supporting this framework, Chui and Leung used the Attachment Style Questionnaire—Short Form in a Chinese population and confirmed that secure attachment is characterized by positive models of self and others, whereas fearful and preoccupied styles are tied to self-doubt and interpersonal vulnerability [37]. Consistently, our patient group also exhibited moderate negative correlations between the perception of others and both fearful (r = −0.336, *p* = 0.01) and dismissive (r = −0.304, *p* = 0.02) styles, while in controls, secure attachment positively correlated with the perception of others (r = 0.467, *p* < 0.0001).

In conclusion, our findings clearly demonstrate that patients with chronic pruritus exhibit significantly higher levels of insecure attachment styles alongside elevated anxiety, depression, and psychosocial burden. Notably, the association between preoccupied attachment and greater itch severity highlights how emotional dysregulation may intensify pruritus symptoms. Thus, attachment disorders seem a to have marked influence on the psychocutaneous and psychosomatic diseases. Modern dermatology increasingly integrates patient-centered and non-invasive approaches that also address the psychosocial burden of skin conditions, as demonstrated in recent studies on aesthetic and inflammatory dermatologic disorders [38]. In light of the limited research directly examining attachment in chronic pruritus, our study provides valuable insight into this overlooked dimension and supports a more integrative, biopsychosocial approach to assessment and treatment.

This study has several limitations. Attachment styles were assessed using self-report instruments, which may be influenced by response bias, and interview-based assessments were not performed. Cultural context may also affect attachment patterns and emotional expression, potentially limiting generalizability beyond the studied population. Additionally, data were collected in 2014; however, attachment styles are relatively stable constructs, and the psychometric tools used remain valid. Importantly, the psychobiological mechanisms underlying chronic pruritus have not fundamentally changed, supporting the continued relevance of the present findings.

Future research should further investigate the longitudinal relationship between attachment insecurity and chronic pruritus to clarify potential causal pathways. From a clinical perspective, routine psychological screening in patients with chronic pruritus—particularly for insecure attachment patterns—may facilitate earlier identification of individuals at risk for heightened symptom severity and psychosocial burden.

## Figures and Tables

**Table 1:** Demographic Characteristics of the Study Groups.

Scales	Itch Severity Low (Mean ± Sd)	Itch Severity Moderate/High (Mean ± Sd) Low (Mean ± Sd)	*p*-Value
GHQ	2.67 ± 2.55	2.57 ± 3.14	0.003 *
HADS—anxiety	6.43 ± 4.15	7.34 ± 4.65	0.003 *
HADS—depression	6.97 ± 3.98	6.33 ± 4.42	0.06
DLQI	9.30 ± 7.66	11.48 ± 1.03	0.0001 *

* indicates statistical significance (*p* < 0.05).

**Table 2:** Relationships between itch severity and GHQ, HADS—anxiety, HADS—depression, DLQI.

Scales	Itch Severity Low (Mean ± Sd)	Itch Severity Moderate/High (Mean ± Sd)	*p*-Value
GHQ	2.67 ± 2.55	2.57 ± 3.14	0.003 *
HADS—anxiety	6.43 ± 4.15	7.34 ± 4.65	0.003 *
HADS—depression	6.97 ± 3.98	6.33 ± 4.42	0.06
DLQI	9.30 ± 7.66	11.48 ± 1.03	0.0001 *

* = *p* < 0.05, statistically significant.

**Table 3:** Impact of itch severity on attachment styles.

Scales	Itch Severity Low (Mean ± Sd)	Itch Severity Moderate/High (Mean ± Sd)	*p*-Value
Fearful attachment	3.56 ± 1.20	3.16 ± 1.22	0.35
Dismissing attachment	3.89 ± 1.00	3.70 ± 1.03	0.73
Secure attachment	3.84 ± 1.13	4.25 ± 1.11	0.44
Preoccupied attachment	3.37 ± 0.87	3.54 ± 0.81	0.01 *

* = *p* < 0.05, statistically significant.

**Table 4:** Scores of attachment styles scale in patient and control group.

Scales	Patient (Mean ± Sd)	Control (Mean ± Sd)	*p*-Value
Fearful attachment	3.71 ± 1.25	2.81 ± 1.02	<0.001 *
Dismissing attachment	3.94 ± 1.08	3.56 ± 0.94	0.041 *
Secure attachment	3.73 ± 1.09	4.56 ± 1.00	<0.001 *
Preoccupied attachment	3.65 ± 0.90	3.35 ± 0.73	0.048 *

* = *p* < 0.05, statistically significant.

**Table 5:** Scores of GHQ, HADS, and DLQI in patient and control group.

Scales	Chronic Pruritus (*n* = 60) (Mean ± Sd)	Control Group (*n* = 60) (Mean ± Sd)	*p*-Value
GHQ	3.98 ± 3.34	1.20 ± 1.71	<0.0001 *
HADS—anxiety	8.23 ± 4.74	6.00 ± 4.03	0.006 *
HADS—depression	8.03 ± 4.35	4.95 ± 3.86	<0.0001 *
DLQI	14.2 ± 9.43	7.67 ± 10.17	<0.0001 *

* = *p* < 0.05, statistically significant.

**Table 6:** Correlation between self–others scores and attachment styles in patient and control group.

	Self		Others	
	Patient	Control	Patient	Control
Fearful	r = −0.492 *p* < 0.0001 *	r = −0.269 *p* = 0.04 *	r = −0.336 *p* = 0.01 *	r = −0.460 *p* < 0.0001 *
Dismissing	r = −0.269 *p* = 0.04 *	r = −0.084 *p* = 0.52	r = −0.304 *p* = 0.02 *	r = −0.246 *p* = 0.06
Secure	r = 0.37 *p* = 0.005 *	r = 0.163 *p* = 0.21	r = 0.389 *p* = 0.001 *	r = 0.467 *p* < 0.0001 *
Preoccupied	r = −0.315 *p* = 0.01 *	r = −0.537 *p* < 0.0001 *	r = 0.166 *p* = 0.20	r = 0.146 *p* = 0.27

* = *p* < 0.05, statistically significant.

## Data Availability

The original contributions presented in this study are included in the article. Further inquiries can be directed to the corresponding author.
